# Nickel salt of phosphomolybdic acid as a bi-functional homogeneous recyclable catalyst for base free transformation of aldehyde into ester†

**DOI:** 10.1039/d0ra04119j

**Published:** 2020-06-09

**Authors:** Anjali Patel, Jay Patel

**Affiliations:** Polyoxometalates and Catalysis Laboratory, Department of Chemistry, Faculty of Science, The Maharaja Sayajirao University of Baroda Vadodara-390002 India anjali.patel-chem@msubaroda.ac.in

## Abstract

A Ni salt of phosphomolybdic acid (NiHPMA) was synthesized and characterized by various physico-chemical techniques such as EDX, UV-Visible spectroscopy, FT-IR, Raman spectroscopy and XPS. FT-IR and Raman spectroscopy confirm the presence of Ni as a counter cation while UV-Visible and XPS studies to confirm the presence of Ni(ii) in the catalyst. The catalyst was evaluated for its bi-functional activity towards the tandem conversion of benzaldehyde to ethyl benzoate and it was found that very small amounts of Ni (2.64 × 10^−3^ mmol) enhance the selectivity towards benzoate. A detailed mechanistic study was carried out by UV-Visible and Raman spectroscopy to confirm that both intermediate species, Mo–peroxo and Ni–oxo, are responsible for higher selectivity towards esters. Further, a study to determine the effect of addenda atoms (heteropoly acid) was also carried out. The catalyst was also found to be viable for a number of aldehydes under optimized conditions.

## Abstract 2

A Ni salt of phosphomolybdic acid (NiHPMA) was synthesized, characterized and evaluated for bi-functional activity *via* conversion of benzaldehyde to ethyl benzoate. The role of Mo-peroxo and Ni-oxo was confirmed by UV-visible/Raman spectroscopy.

## Graphical abstract

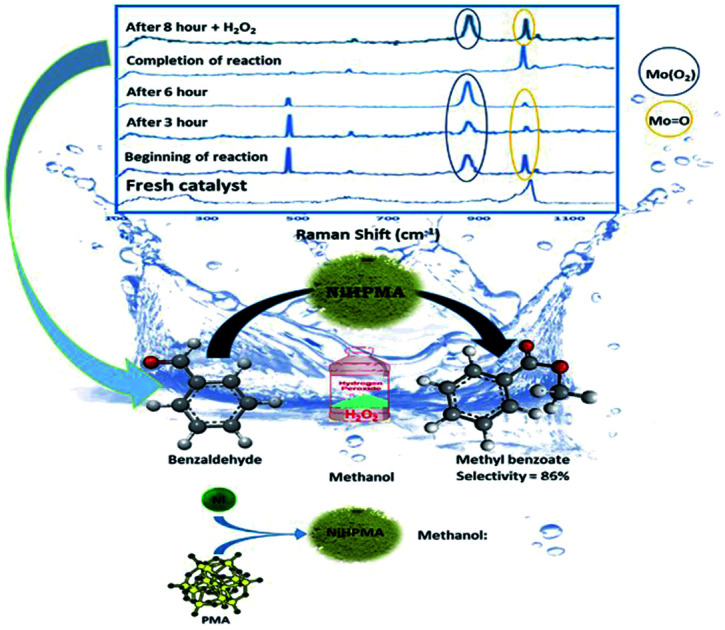

## Introduction

Keggin type heteropoly acids (HPAs) are an exclusive class of inorganic compounds which have metal oxygen supra-molecular clusters of early transition metal groups V (V, Nb, and Ta) and VI (Cr, Mo, and W) in their higher oxidation states^1–3^ and have an oxo-enriched surface, high thermal stability, and tunable acidic/redox properties.^4–9^ They are useful acids and oxidation catalysts in various reactions because their catalytic features can be varied at a molecular level.^10–16^ In particular, the use of HPAs and their related compounds as green catalysts is a field of increasing worldwide importance and numerous studies have been carried out in basic and applied research in the last four decades.^17–22^ For example, heteropoly acid-based ionic liquids (HPAILs) as novel functionalized ionic liquids have attracted increasing attention,^23,24^ and have been reported as efficient and recyclable catalysts for lipophilic alkene epoxidation and alcohol selective oxidation with aqueous H_2_O_2_.^25–28^

Recently, the development of multicomponent reactions (MCRs) and their applications for one-pot production of several important compounds has fostered the synthetic toolbox expansion.^29–32^ Although MCRs have been regarded as “advanced tools for sustainable organic synthesis”, many have questioned their potential due to several drawbacks such as low yields, long reaction times, harsh conditions, requirement of reagents excesses, reproducibility issues, and others.^33,34^ Towards the same, HPAs-catalysed reactions have gained considerable interest for the synthesis of few important compounds.^20,35^

**Scheme 1:**
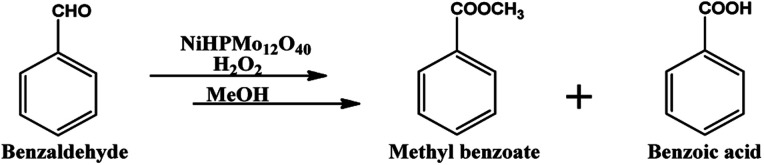
Oxidative esterification of benzaldehyde.

More recently it was found that HPA derivatives embedded in ionic liquids media have been successfully applied to catalyse some MCRs.^36–38^ For example, a polymeric heteropolyacid-containing pyridinium IL catalyst proved to be a promising system for the multicomponent Biginelli reaction.^39^ The Biginelli reaction is believed by some to be the most important MCR.^40^ This MCR (Scheme 1) allows the direct synthesis of bioactive DHMPs (3,4-dihydropyrimidin-2(1*H*)-ones or -thiones) such as monastrol, piperastrol, and enastron.^41–46^

It is well known that HPAs possess a very strong acidity in form of protons, which can be changed by varying the chemical composition of the primary structure of the heteropolyanion.^17^ These exchangeable protons can be easily replaced by other cations, without affecting primary structure.^47,48^ Depending on the nature of cations (*i.e.* salt of HPAs), acidic/redox properties can be tuned, which make them important catalytic materials for various organic transformations.^49–51^ Amongst, phosphotungstic acid is studied in detailed and number of reports are available for the same. At same time comparatively studied on phosphomolybdic acid is very less.

In 1974 Tsigdinos,^52^ first time, reported the synthesis of Ni_3_[PMo_12_O_40_]_2_·34H_2_O and studied their thermal behavior, solubility and hydrolytic strength in different solvents mixture including aqueous medium. After almost two decades, in 1994, Mizuno *et al.*^53^ reported synthesis of Cs_2.5_Ni_0.08_H_0.34_PMo_12_O_40_ and its use for oxidation of isobutene to methacrylic acid and methacrolein using molecular O_2_. In 1999, Demirel *et al.*^54^ reported liquefaction of Wyodak coal using same catalyst, reported by Tsigdions.^52^ Later, in 2010, Rabia *et al.*^55^ reported synthesis of (NH_4_)_*x*_M_*y*_H_*z*_PMo_12_O_40_ (M = Ni^2+^, Co^2+^ or Fe^3+^) using same method as Mizuno *et al.* reported^53^ and catalytic activity for oxidation of propane. In 2015, the same group came up with the use of soluble H_3−2*x*_Ni_*x*_PMo_12_O_40_ and (NH_4_)_3−2*x*_Ni_*x*_PMo_12_O_40_ where *x*: 0.25–1.5 for oxidation of cyclohexanone to adipic acid.^56^ Further in 2019, same group^57^ synthesized a series of transition metal salt of phosphomolybdic acid [HMPMo_12_O_40_ (M: Co, Ni, Mn, Cu or Zn)] by using their own method and described their use as catalysts for the synthesis of adipic acid from cyclohexanone.

Above literature survey shows (i) few reports are available on MCRs using HPAs based compounds, in other words combination of HPAs and ionic liquids have been used ony for MCRs (ii) oxidative esterification was not at all studied (iii) although nickel salt of phosphomolybdic acid is excellent and sustainable for the oxidation reaction, full characterization of the same is missing and no single report is available for any tandem reaction to show the bi-functional effect of Ni as well as Mo.

Oxidative esterification is one of the best example of tandem reaction, in which both Lewis acidity as well as redox ability of the catalyst plays an important role. Oxidative esterification of benzaldehyde is much more attracting due to the importance and use of methyl benzoate for perfumery, food industry, pharmaceutical, agrochemical and natural products.^58,59^

In the present work, for the first time, we report detailed characterization of Ni salt of phosphomolybdic acid by EDX, TGA, UV-Vis, FT-IR, Raman spectroscopy, and XPS along with catalytic evaluation for tandem oxidative esterification of benzaldehyde to ester. The reaction was carried out using methanol and H_2_O_2_ and various reaction parameters such as catalyst amount, amount of H_2_O_2_, the volume of methanol, reaction time and reaction temperature were optimized for maximum conversion as well as selectivity towards ester. The recycling and reusability of synthesized catalyst was carried out up to three cycles. Detailed reaction mechanism was proposed based on Raman and UV-Visible spectroscopy studies.

## Experimental

### Materials

All chemicals used were of A. R. grade. Phosphomolybdic acid (H_3_PMo_12_O_40_), nickel acetate, benzaldehyde, methanol, 30% hydrogen peroxide (H_2_O_2_) v/v and dichloromethane obtained from Merck were used as received.

### Synthesis of nickel salt of phosphomolybdic acid (NiHPMA)

The synthesis was carried out by the following reported method in literature.^60^ A saturated solution of nickel acetate (0.1244 g, 0.5 mmol) was added drop wise into solution of H_3_PMo_12_O_40_ (0.9126 g, 0.5 mmol) dissolved in a minimum amount of water. The resulting mixture was aged for 1 h at 80 °C and excess of water was evaporated on water bath. The obtained material was dried at 100 °C for 10 hours and calcined at 300 °C under air atmosphere for 2 h in a muffle furnace. The resulting green color, material was designated as NiHPMA.

To see any decomposition, HPMA was also calcined under the same conditions and it was found that color remains yellow, good agreement with the reported literature.^61^

### Characterization

Elemental analysis was performed by EDX using JSM 5610 LV combined with INCA instrument for EDX-SEM. Thermo-gravimetric analysis (TGA) was performed under nitrogen atmosphere conditions on the Mettler Toledo star SW 7.01 up to 500 °C. The UV-Vis spectra were recorded at room temperature on the Perkin Elmer 35 LAMDA instrument using a quartz cell of 1 cm within a range of 200–1100 nm. FT-IR spectra were carried out using Shimadzu instrument (IRAffinity-1S) with KBr wafer pellets. The Fourier Transform Raman (FT-Raman) spectra were performed on a FT-Raman spectrophotometer (Model Bruker FRA 106). Measurement of X-ray photoelectron spectroscopy (XPS) was performed with the PHI 5000Versa prob II device with Auger electron spectroscopy (AES). Philips diffractometer instrument (Model Pw-1830) was used to perform the powder X-ray diffraction (Powder XRD).

### Catalytic evaluation

For oxidative esterification of benzaldehyde, 50 mL glass batch reactor provided with a double walled air condenser, magnetic stirrer and guard tube, was charged with benzaldehyde (10 mmol) with H_2_O_2_ (30 mmol, 30% H_2_O_2_ v/v), methanol (7 mL), catalyst (5 mg) and stirred at 80 °C for 6 h. After completion of reaction, mixture was extracted with dichloromethane. The products in obtained organic phase were analyzed by GC keeping the following temperature program: initially, the oven and capillary temperature was set at 80 °C and 250 °C respectively, then the temperature increased from 80 to 240 °C, with a rate of 10 °C min^−1^, compared with the reference standard samples and no overlapping was found. For reader's convenience, GC profile for the authentic samples as well as reaction mixture are included in the ESI (Fig. S1).† TON over number calculated by following formula: 
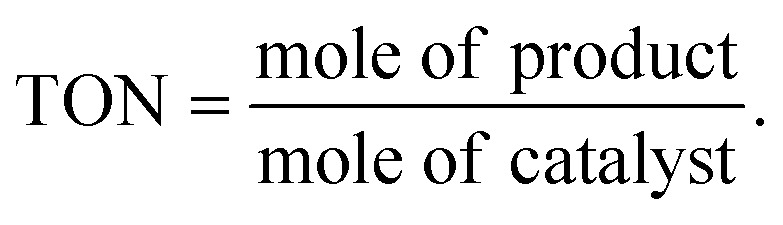


### Procedure for recycling of the used catalyst

As recycling of 5 mg catalyst is difficult, we have kept three sets of reaction under same optimized experimental conditions. After completion of reaction, the reaction mixture was cooled to room temperature and organic layer was extracted by dichloromethane (DCM). The aqueous phase was dried at 100 °C to recover the homogeneous solid catalyst. The obtained solid catalyst (recycled catalyst) was used for next cycle. The similar procedure was followed up to three cycles.

## Results and discussion

### Characterization of catalyst

**Fig. 1:**
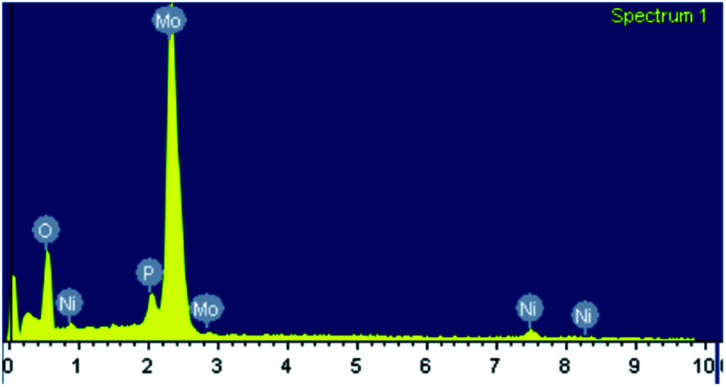
EXD mapping of NiHPMA.

The EDX mapping of NiHPMA in Fig. 1 shows that presence of all expected elements. The ascertained EDX values of all the elements were in good agreement with the theoretical values. EDX value (% wt): Ni, 2.89; Mo, 57.15; P, 1.39; O, 38.57 calculated value (% wt): Ni, 2.92; Mo, 57.34; P, 1.54; O, 38.20.

TGA of NiHPMA shows (Fig. S3†) initial weight loss of 2.46% up to 100 °C, due to the removal of adsorbed water. Further, it shows 4.11% weight loss up to 280 °C corresponding to removal of water of crystallization. Further, no appreciable weight loss was noticed, indicating the thermal stability of the synthesized material up to 500 °C. Based on the total weight loss, the number of water molecule was calculated using the formula
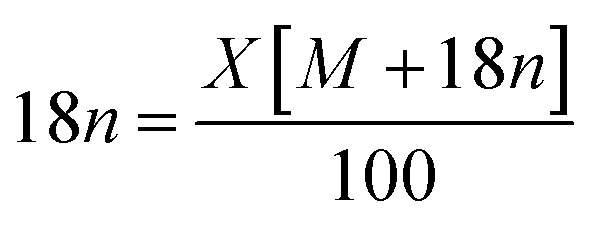
where, *n* = number of water molecules, *X* = % loss from TGA and *M* = molecular weight of substance (without water of crystallization) was found to be 6. From the EDX and TGA, the chemical formula of the synthesized material is proposed NiH[PMo_12_O_40_]·6H_2_O.

The UV-Visible spectrum of NiHPMA shows (Fig. S4†) the absorption peak at 230 nm due to the charge transfer between O^2−^ → Mo^6+^.^62^ In Addition, it also shows an absorption at 396 nm, corresponding to the presence of Ni(ii),^58^ first indicates the presence of Ni in the synthesized materials.

**Fig. 2:**
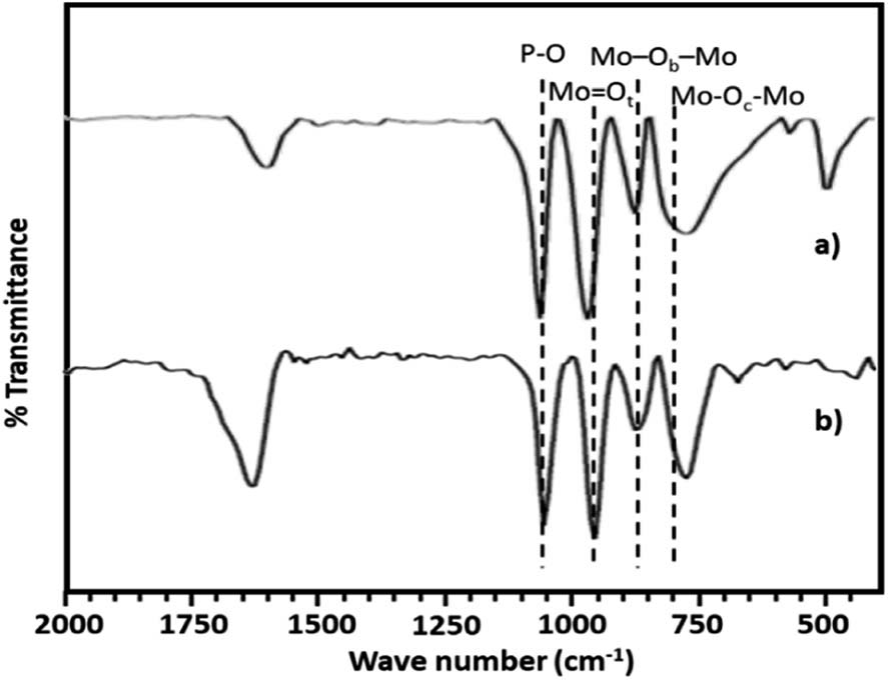
FT-IR spectra of (a) PMA and (b) NiHPMA.

The FT-IR spectra of PMA shows (Fig. 2a) the characteristic bands at 1070, 965, 870 and 790 cm^−1^ corresponding to stretching vibration of P–O, Mo=O_t_, Mo–O_b_–Mo and Mo–O_c_–Mo respectively.^63,64^ Whereas NiHPMA shows all characteristic bands without any significant shift, 1070, 960, 871 and 786 cm^−1^ corresponding to stretching vibrations of P–O, M=O_t_, Mo–O_b_–Mo and Mo–O_c_–Mo respectively with an additional band at 455 cm^−1^, characteristic of (Ni–O) bond indicating the presence of Ni.^56,57^ Moreover, the presence of P–O stretching is at exactly same wavelength (1070 cm^−1^) confirms the presence of Ni as counter cation only, *via* exchange of available protons of PMA.

**Fig. 3:**
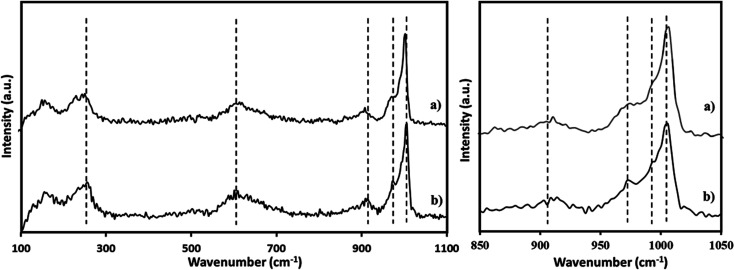
Raman spectra of (a) PMA (b) NiHPMA.

The Raman spectra of PMA was recorded (Fig. 3a) and is in good agreement with the reported one.^65,66^ The Raman spectra of NiHPMA shows (Fig. 3b) peaks with slight shifting at 1004, 980, 973, 913, 609, and 295 cm^−1^ for *ν*_s_(Mo=O_t_), *ν*_as_(Mo–O_t_), *ν*_s_ (P–O), *ν*_as_(Mo–O_b_–Mo), *ν*_s_(Mo–O_b_–Mo) and *ν*_s_(Mo–O_a_) vibration respectively.^67^ The observed shifting in all the characteristic bands may be due to change in the environment because of the exchange of available protons of PMA by Ni.

**Fig. 4:**
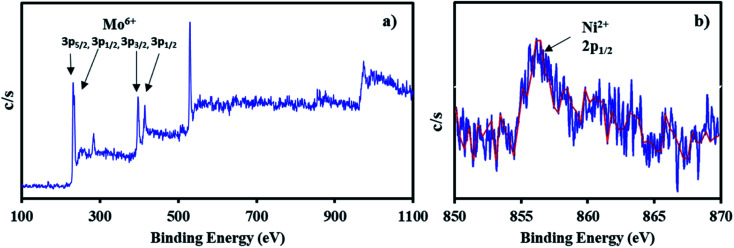
X-ray photoelectron spectrum of NiHPMA in which (a) Mo(vi) and (b) Ni(ii).

The XPS of NiHPMA shows (Fig. 4.) that intense peaks at 233, 397 and 416 eV corresponds to 3d_3/2_, 3p_3/2_ and 3p_1/2_ respectively and low intense peak at 235 eV corresponds to 3d_3/2_ energy level of Mo(vi).^68,69^ Furthermore, the peak at 871 eV with lower intensity corresponds to 2p_1/2_ energy level of Ni(ii) in good agreement with reported ones.^70–72^ However, low intensity of peak of Ni can be attributed to low concentration of Ni (2.64 × 10^−3^ mmol) in the synthesized material.

### Catalytic activity

Oxidative esterification was selected as a model reaction for evaluation of catalyst efficiency and for this benzaldehyde and methanol were used as test substrates in the presence of hydrogen peroxide as an oxidant (Scheme 1). The effect of various reaction parameters such as catalyst amount, methanol volume, substrate to H_2_O_2_ ratio, time and temperature were studied to optimize the conditions for maximum conversion as well as selectivity towards the ester. Each experiment was carried out thrice and the results obtained were reproducible with an error of ±1–1.5%.

To evaluate the effect of H_2_O_2_, the reaction was carried out with different mole ratio of benzaldehyde to H_2_O_2_ by keeping all other parameters constant. Obtained result shows (Table 1) that on increasing the amount of oxidant from 10 mmol (1 : 1) to 40 mmol (1 : 4), conversion also increases which is in good agreement with chemical dynamics,^72,73^ indicate that the oxidative esterification could be improved by increasing the amount of H_2_O_2_. Concurrently, the selectivity towards desired product, methyl benzoate, decreases because the higher amount of oxidant will tolerate more oxidation of benzaldehyde to convert into benzoic acid. Hence 1 : 3 ratio of substrate to H_2_O_2_ was optimized.

**Table 1:** Effect of mole ratio^a^

Substrate : H_2_O_2_	% Conversion	% Selectivity	
		Ester	Acid
1 : 1	51	96	4
1 : 2	62	88	12
1 : 3	68	86	14
1 : 4	74	64	36

a Reaction conditions: catalyst (5 mg), catalyst/substrate ratio (2.64 × 10^−4^) benzaldehyde (10 mmol), oxidant (30% H_2_O_2_ v/v), methanol (7 mL), temp. (80 °C) and time (6 h).

**Fig. 5:**
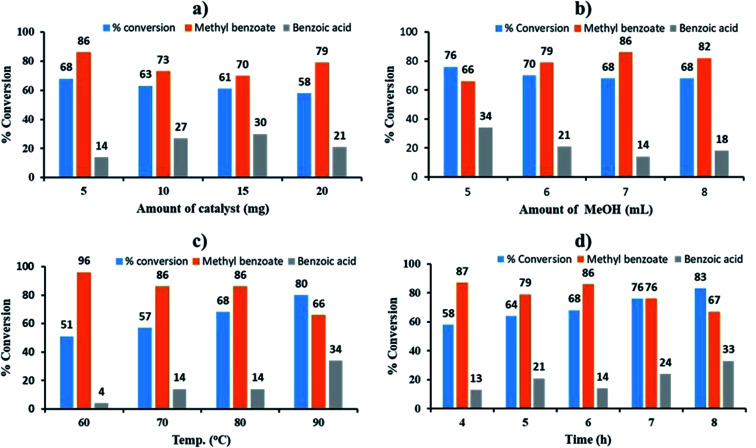
Optimization of parameters for oxidative esterification of benzaldehyde. Reaction condition: (a) effect of catalyst amount: benzaldehyde (10 mmol), H_2_O_2_ (30 mmol, 30% H_2_O_2_ v/v), methanol (7 mL), temperature (80 °C) and time (6 h); (b) effect of methanol: catalyst (5 mg), catalyst/substrate ratio (2.64 × 10^−4^), benzaldehyde (10 mmol), H_2_O_2_ (30 mmol, 30% H_2_O_2_ v/v), temperature (80 °C) and time (6 h); (c) effect of temp.: catalyst (5 mg), catalyst/substrate ratio (2.64 × 10^−4^), benzaldehyde (10 mmol), H_2_O_2_ (30 mmol, 30% H_2_O_2_ v/v), methanol (7 mL) and time (6 h); (d) effect of time: catalyst (5 mg), catalyst/substrate ratio (2.64 × 10^−4^), benzaldehyde (10 mmol), H_2_O_2_ (30 mmol, 30% H_2_O_2_ v/v), methanol (7 mL) and temperature (80 °C).

The effect of catalyst amount was studied by varying the amount from 5 to 20 mg. The obtained results are shown in Fig. 5a. On increasing the amount of catalyst from 5 to 20 mg, decrease in % conversion as well as % selectivity of ester was observed due to the unproductive decomposition of H_2_O_2_ in the presence of excess catalyst which generates additional water, shifting the equilibrium towards left and hence the decrease in conversion, which is in good agreement with the reported one.^59^ Therefore 5 mg of catalyst amount was optimized for maximum 68% conversion with highest selectivity (86%) towards ester.

The effect of methanol amount was investigated in the range of 4 to 8 mL (Fig. 5b). Obtained results showed that on increasing volume of methanol, % conversion decreased and selectivity of ester increased up to 7 mL, which may be due to dilution of substrate with methanol. Hence, 7 mL of methanol was optimized from the obtained results.

The effect of temperature was screened between the range of 60 to 90 °C. The obtained results show (Fig. 5c) that on increasing reaction temperature up to 90 °C, conversion increased and the selectivity towards ester decreased because of thermal and catalytic decomposition of H_2_O_2_ at high temperature.^59,72^ Therefore, 80 °C was optimized.

The effect of reaction time was monitored between range of 4 to 8 h (Fig. 5d). Obtained results display that as reaction time increases, conversion also increases but the selectivity of ester decreases which is due to decomposition of H_2_O_2_ generated water molecule, which leads to hydrolysis of ester to aldehyde.

The optimized condition for maximum 68% conversion with highest 86% selectivity are: benzaldehyde, 10 mmol; 30% H_2_O_2_ v/v, 30 mmol; catalyst, 5 mg (active amount of Ni: 0.155 mg; catalyst/substrate ratio, 2.64 × 10^−4^); methanol, 7 mL; temperature, 80 °C; and time, 6 hours, TON: 2576.

### Control experiment and investigation of mechanism

In control experiments, the reaction was carried out with Ni(CH_3_COO)_2_ and PMA in optimized experimental conditions and obtained results are shown in Table 2. In case of Ni(CH_3_COO)_2_, 49% conversion was obtained with 64% selectivity of ester because of the Lewis acidity of Ni, whereas in case of PMA, 46% conversion was achieved with 67% ester selectivity. Furthermore, when the reaction was carried out with NiHPMA, 68% conversion with 86% selectivity towards ester was achieved due to Ni increased Lewis acidity and total acidity of catalyst which enhance selectivity of ester. The obtained result indicates the bi-functional nature of catalyst where Mo contributes for oxidation and Ni for esterification due to its Lewis acidity.

**Table 2:** Control experiment for oxidative esterification of benzaldehyde

Catalyst	% Conversion	% Selectivity	
		Ester	Acid
aNi(CH_3_COO)_2_	49	64	36
PMA	46	67	33
aNiHPMA	68	86	14
aNiHTPA	45	97	03

a Reaction conditions: catalyst (5 mg), catalyst/substrate ratio (2.64 × 10^−4^), benzaldehyde (10 mmol), oxidant (30 mmol, 30% H_2_O_2_ v/v), methanol (7 mL), temp. (80 °C) and time (6 h). Active amount of nickel (0.155 mg).

In order to see the effect of an addenda atom, the catalytic activity of Ni salt of phosphotungstic acid (NiHTPA) was also evaluated for the said reaction under same experimental conditions and obtained results are presented in table. It is very interesting to note down that the trend is exactly follows the know order of acidity and redox ability of different heteropolyacids.

Order of acidity: phosphotungstic acid (PW_12_) > silicotungstic acid (SiW_12_) > phosphomolybdic acid (PMo_12_) > silicomolybdic acid (SiMo_12_).

Order of redox ability: phosphomolybdic acid (PMo_12_) > silicomolybdic acid (SiMo_12_) > silicotungstic acid (SiW_12_) > phosphotungstic acid (PW_12_).

As the PMo_12_ has maximum oxidation ability, it will give more conversion (as per the proposed mechanism, Mo–peroxo is responsible for oxidation), while being a most acidic PW_12_ will contribute more towards the formation of the ester in addition to Ni (Lewis acidity).

To know the role of each substrate (Table 3), various experiments were carried out: (i) without catalyst (benzaldehyde + methanol + H_2_O_2_) the reaction proceed to give negligible conversion, which indicates the requirement of efficient catalyst for the fast reaction (ii) without H_2_O_2_ (catalyst + methanol + benzaldehyde) the reaction did not show significant conversion (iii) without methanol (catalyst + benzaldehyde + H_2_O_2_), gives 100% conversion with single selective product benzoic acid. This study indicates that all three components; catalyst, methanol and H_2_O_2_ are essential for the oxidative esterification reaction.

**Table 3:** Role of substrates^a^

Catalyst	% Conversion	% Selectivity	
		Ester	Acid
Without catalyst (benzaldehyde + methanol + H_2_O_2_)	4	—	100
Without H_2_O_2_ (catalyst + methanol + benzaldehyde)	15	76	24
Without methanol (catalyst + benzaldehyde + H_2_O_2_)	45	—	100

a Reaction conditions: catalyst (5 mg), catalyst/substrate ratio (2.64 × 10^−4^), benzaldehyde (10 mmol), oxidant (30 mmol, 30% H_2_O_2_ v/v), methanol (7 mL), temp. (80 °C) and time (6 h).

To confirm either the reaction proceeds *via* formation of acetal as intermediate according to a reported general reaction mechanism^37^ or through benzoic acid as intermediate, earlier reported by our group^58,72^ the following set of reactions were carried out: (i) when benzoic acid was used as a substrate under optimized condition (in absence of H_2_O_2_) yielding 45% conversion with 100% selectivity of methyl benzoate. (ii) in another set of reaction, benzaldehyde was allowed to react with methanol in presence of catalyst and absence of H_2_O_2_ yielding 15% conversion with 76% selectivity of ester and 24% selectivity of benzoic acid. The reaction was prolonged for 3 h with addition of H_2_O_2_, yielding 50% conversion as well as 69% selectivity of ester. This study shows that oxidative esterification reaction proceeds through *in situ* generation of benzoic acid rather than an acetal intermediates.

**Fig. 6:**
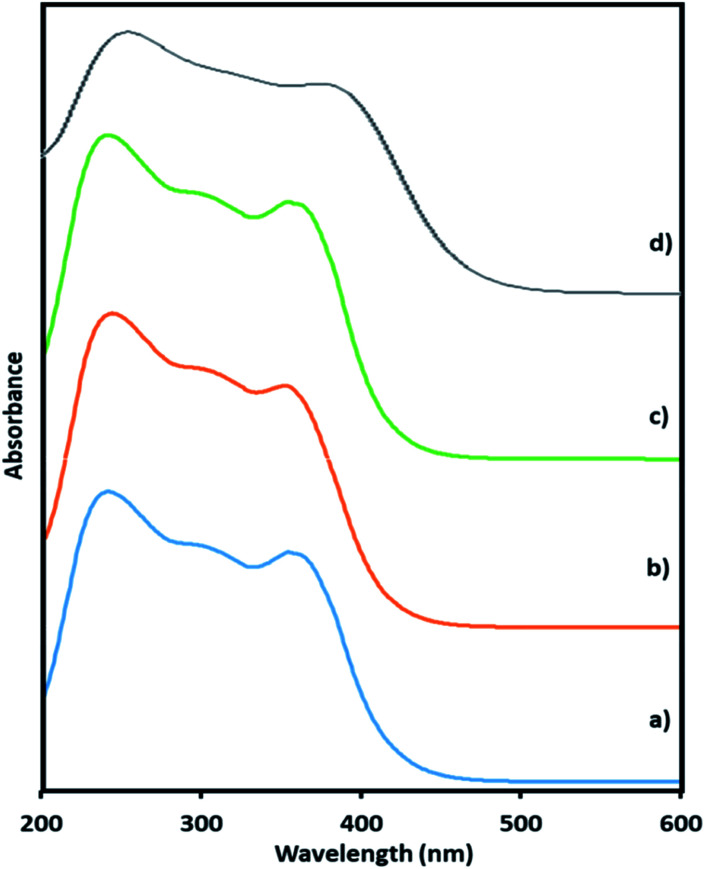
UV-Vis spectra of (a) reaction starting point (b) after 3 h (c) after 6 h (d) after 8 h.

**Fig. 7:**
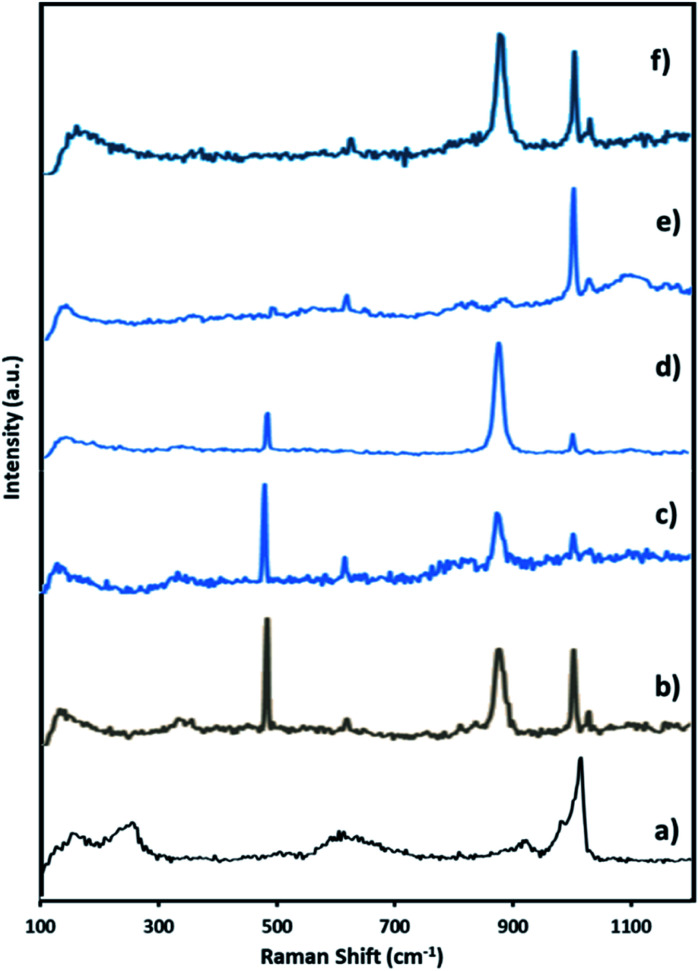
Raman spectra of (a) fresh NiHPMA (b) reaction starting (c) after 3 h (d) after 6 h (e) after 8 h and (f) after 8 h + H_2_O_2_.

Here, in this paper, first time we attempted for more insight to understand the mechanistic aspects and for that the said reaction was monitored at different time intervals by UV-Vis and Raman spectroscopy (Fig. 6 and 7). UV-Vis spectra of fresh NiHPMA shows (Fig. S2†) two absorbance peaks at 230 and 396 nm due to the ligand to metal charge transfer (O^2−^ → Mo^6+^) and d–d transition of Ni respectively.^58,75^ It is interesting to note that the formation of new absorbance peaks at 363 and 309 nm (Fig. 6a) after the addition of H_2_O_2_ indicates presence of oxo species of Ni(O_2_)^58^ and peroxo species of {PO_4_[MoO(O_2_)_2_]_4_}^3–75^ respectively, which are responsible for catalytic activity and are in good agreement with the reported one. UV-Vis spectrum were taken after 3 h (Fig. 6b) as well as after 6 h (Fig. 6c) and was found similar confirming the presence of active species. While after the completion of 8 hours the disappearance of absorbance bands at 365 and 309 nm and reappearance of original peaks (Fig. 6d) indicate that both, Ni and Mo play role for the mentioned reaction.

**Fig. 8:**
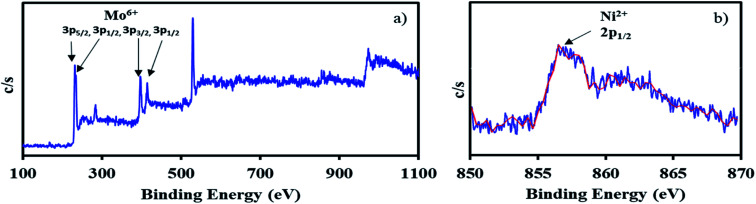
XPS of recycle NiHPMA in which (a) Mo(vi) and (b) Ni(ii).

**Scheme 2:**
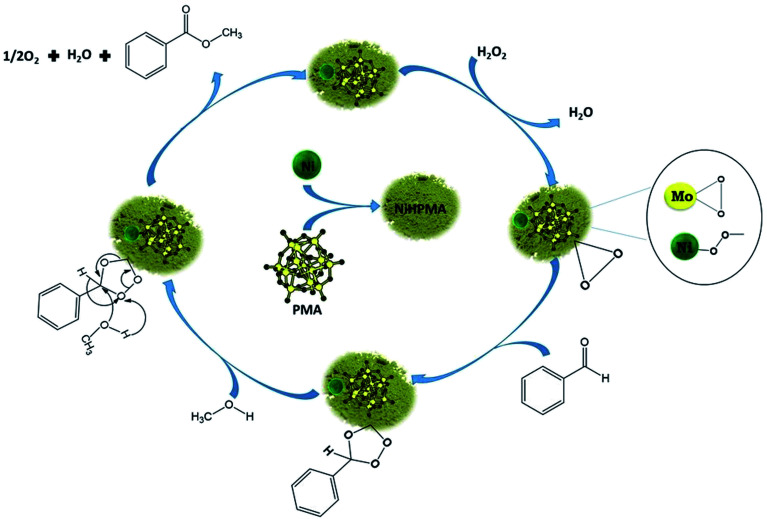
Proposed reaction mechanism for oxidative esterification of benzaldehyde.

Further to confirm the above results, same reaction mixtures were studied by Raman spectroscopy. Unfortunately, we could not get any information about Ni oxo species due to very less concentration of active Ni (2.64 × 10^−3^ mmol). However, the study clearly shows that peroxo species of Mo played role for *in situ* oxidation of benzaldehyde to benzoic acid. Fig. 7. Shows the Raman spectra of NiHPMA at different time interval during the reaction. After the addition of H_2_O_2_, a new peak appears at 881 cm^−1^ (Fig. 8b), which is a characteristic of Mo(O_2_)^62,75^ with a less intense and slight shifting showing the formation of peroxo species immediately after addition of H_2_O_2_. With the progress of the reaction the intensity of the Mo=O (1004 cm^−1^) peak decreases (Fig. 7c and d) with increase in the intensity of the Mo(O_2_) band due to the more formation of peroxo species. After completion of the reaction (Fig. 7e), the original band of 1004 cm^−1^ (Mo=O) reappears. Further, in order to confirm the formation of peroxo species, H_2_O_2_ was once again added into the reaction mixture and we found the formation of a new band at 881 cm^−1^ (Fig. 7f). Thus, the study confirms the formation of peroxo species during the reaction is because of H_2_O_2_. The following mechanism (Scheme 2), all most same as reported by our group earlier.^58,72^ It is known that for oxidation reactions involving polyoxometalates and transition metals, H_2_O_2_, first binds to the metal centre and then transfers an oxygen atom to the substrate. Thus, the activation of the metal centre results *via* the generation of the active species, peroxo species.^76^ In the present case also, the formation of active metal–peroxo intermediate (where, metal is both, Mo as well as Ni) (Fig. 6 and 7) is confirmed by UV-Vis and Raman spectroscopy studies. In the beginning of the reaction, NiHPMA reacts with 30% H_2_O_2_ (v/v) to produce the active metal–peroxo intermediate-II, which is an active species for the insertion of benzaldehyde to form intermediate-III. Finally, MeOH directly reacts with intermediate-III and form product (methyl benzoate) as shown in Scheme 2 (step-IV). It can be concluded that both Mo and Ni contributes simultaneously in terms of Mo–peroxo species for oxidation while Ni–oxo species for esterification reaction.

### Recycling of catalyst

The obtained results, with the regenerated catalyst in optimized identical conditions, show (Table 4) no significant change in % conversion as well as % selectivity of ester up to three cycles. Obtained result indicates that during the reaction the catalyst remains stable, and can be reused over multiple cycles.

**Table 4:** Recycling study^a^

Catalyst	% Conversion	% Selectivity	
		Ester	Acid
Fresh	68	86	14
Cycle – 1	67	84	16
Cycle – 2	67	84	16
Cycle – 3	67	84	16

a Reaction conditions: catalyst (5 mg), catalyst/substrate ratio (2.64 × 10^−4^), benzaldehyde (10 mmol), oxidant (30 mmol, 30% H_2_O_2_ v/v), methanol (7 mL), temp. (80 °C) and time (6 h).

### Characterization of regenerated catalyst

Regenerated catalyst was characterized by EDX, XPS, FT-IR and UV-Visible for the confirmation of prevention of catalyst structure.

EDX values of regenerated NiHPMA (% wt): Ni, 2.87; Mo, 57.16; P, 1.37; O, 38.60 is in good agreement with EDX values of fresh catalyst, indicating no leaching of Ni during the reaction.

The X-ray photoelectron spectra of recycled NiHPMA is shown in Fig. 8. The obtained spectrum show intense peaks at 234, 236, 397 and 416 eV which correspond to 3d_5/2_, 3d_3/2_, 3p_3/2_ and 3p_1/2_ energy level of Mo(vi)^68,69^ and a low intense peak at 856 eV which corresponds to 2p_1/2_ of Ni(ii)^70–72^ on the surface and are identical with fresh catalyst NiHPMA. This shows that the catalyst structure remains intact even after regeneration.

**Fig. 9:**
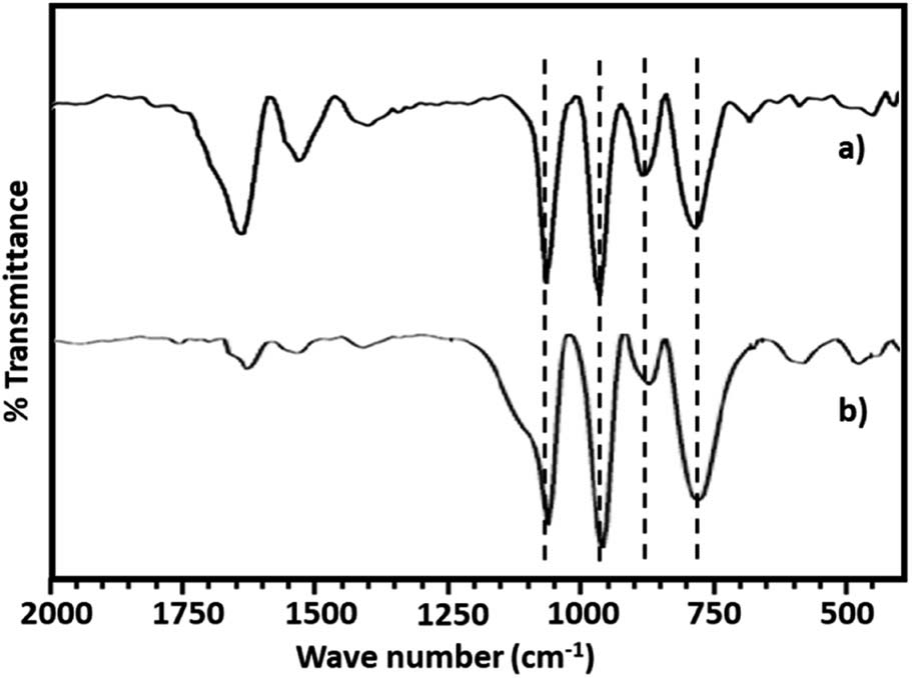
FT-IR spectra of (a) fresh NiHPMA (b) recycle NiHPMA.

The FT-IR spectra of regenerated NiHPMA show (Fig. 9) bands at 1065, 964, 879, 787 and 456 cm^−1^ corresponding to *ν*_as_(P–O), *ν*_as_(M=O_t_), *ν*_as_(Mo–O_b_–Mo), *ν*_as_(Mo–O_c_–Mo) and *ν*_as_(Ni–O) respectively, which is similar to the fresh catalyst without exhibiting any significant shift. This indicates that the catalyst structure is intact even after regeneration. However, in terms of intensity, spectrum differs slightly from the fresh one, which may be due to the sticking of the substrates on the surface of regenerated catalyst.

**Fig. 10:**
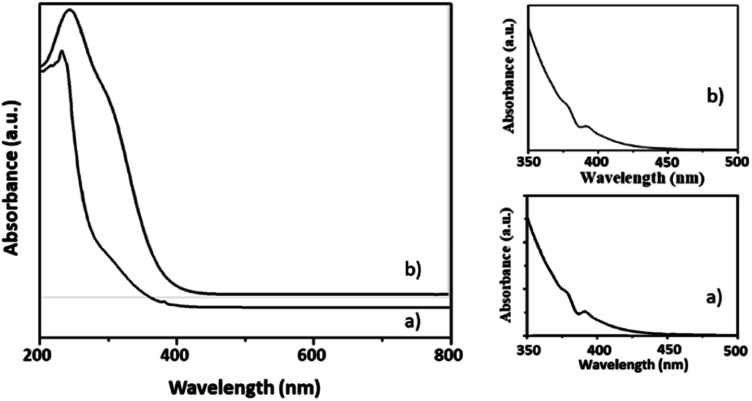
UV-vis spectra of the (a) Fresh NiHPMA and (b) recycle NiHPMA.

The UV-Vis spectra of regenerated catalyst was carried in aqueous phase and shows (Fig. 10) d–d transition at 397 nm characteristic peaks for Ni(ii).^58^ Also LMCT charge transfer between O and Mo at 230 nm was observed,^62^ which is identical with fresh catalyst and confirms that the Keggin unit remains intact.

### Effect of different aldehyde substrate

To find reactivity of synthesized catalyst towards different aromatic aldehyde, the catalytic activity was carried out under optimized reaction condition and obtained results are shown in Table 5. Study shows that the strong electron withdrawing group –Cl facilitates the reaction more as compared to –Br as expected. Aliphatic aldehyde also gives satisfactory conversions. This achieved result displays that the activity of synthesized catalyst with various substrates under mild reaction condition is outstanding.

**Table 5:** Substrate study^a^

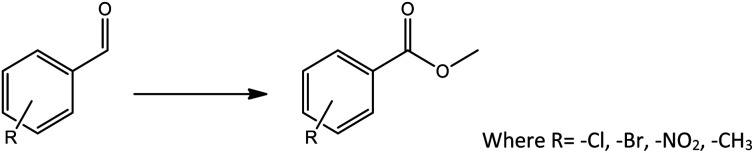				
Sr.	Substrate	Product	% Conversion/% selectivity	TON
1	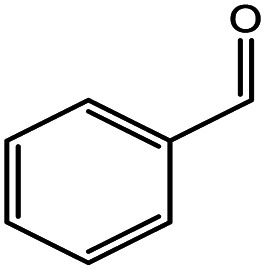	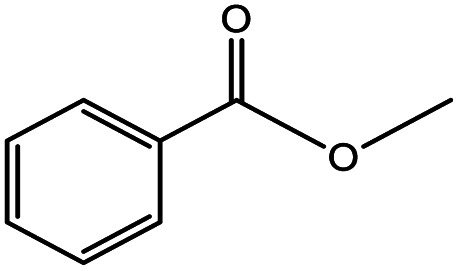	68/86	2576
2	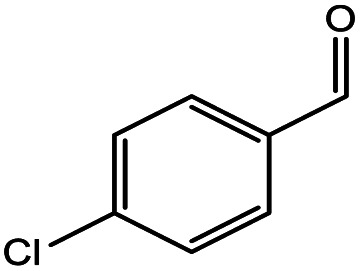	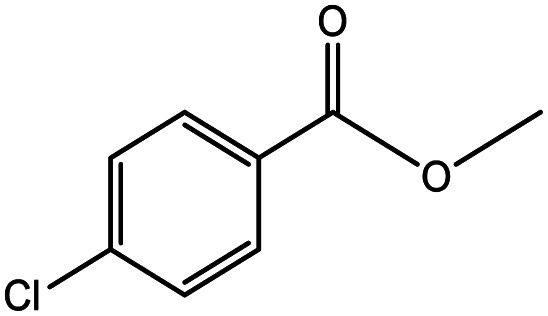	72/88	2711
3	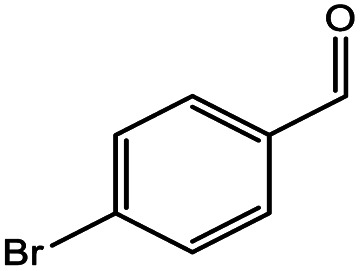	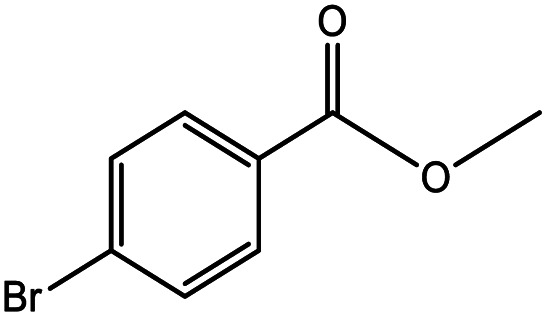	69/87	2590
4	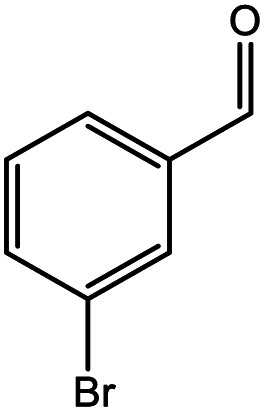	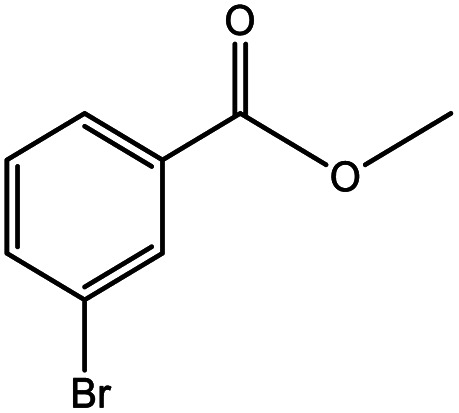	65/86	2447
5	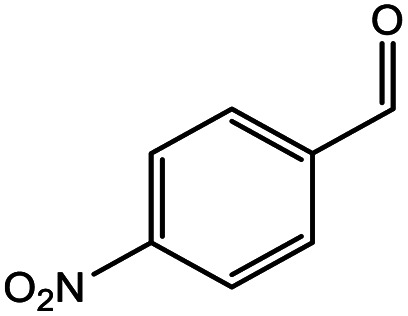	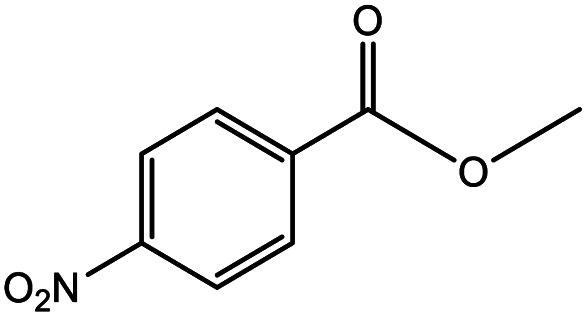	67/89	2522
6	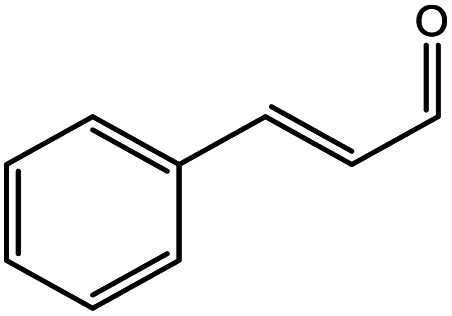	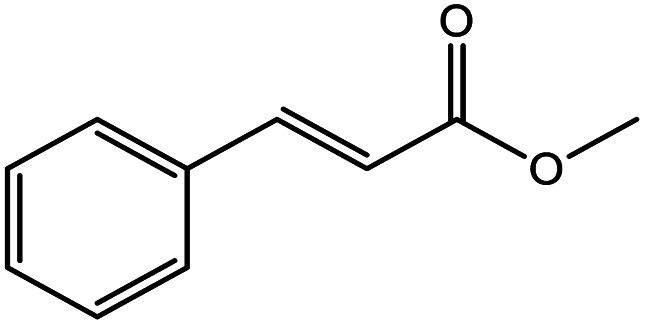	61/81	2296
7	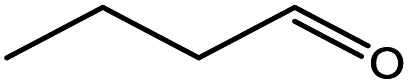	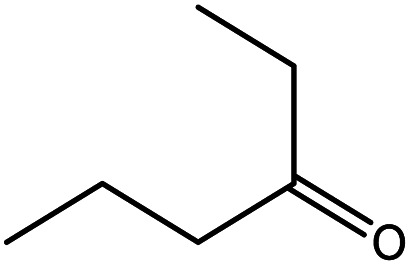	59/77	2221

a Reaction condition: catalyst amount (5 mg), catalyst/substrate ratio (2.64 × 10^−4^), substrate (10 mmol), H_2_O_2_ (30 mmol, 30% H_2_O_2_ v/v), methanol (7 mL), temperature (80 °C) and time (6 h).

## Conclusions

First time we have come up with the bi-functional catalytic activity of Ni (Lewis acid) and Mo (redox property) for conversion of benzaldehyde to benzoate. Very small amount of Ni (0.155 mg) can enhance TON from 1731 to 2560. The catalyst is found to be effective for number of aldehydes, giving % selectivity of 77 to 89% of ester with maximum 2711 TON. Even though the catalyst is homogeneous, we can recycle by simple method and reuse for a number of cycles. Formation as well as role of intermediates, peroxo and oxo species, were confirmed by UV-Visible and Raman spectroscopy.

## Conflicts of interest

There are no conflicts to declare.

## Supplementary Material

RA-010-D0RA04119J-s001
