# Supplementary material for: Nickel salt of phosphomolybdic acid as a bi-functional homogeneous recyclable catalyst for base free transformation of aldehyde into ester
Source: RSC Adv. 2020 Jun 9;10(37):22146–55. doi: 10.1039/d0ra04119j (PMC9054555; doi:10.1039/d0ra04119j)
Supplement: RA-010-D0RA04119J-s001 [file RA-010-D0RA04119J-s001.pdf]

Electronic Supplementary Material (ESI) for RSC Advances.
This journal is © The Royal Society of Chemistry 2020

Supplementary Information

Nickel salt of Phosphomolybdic acid as a bi-functional homogeneous recyclable catalyst for base free transformation of aldehyde into ester

Anjali Patel* and Jay Patel

***Polyoxometalates and Catalysis Laboratory, Department of Chemistry, Faculty of Science, The Maharaja Sayajirao University of Baroda, Vadodara-390002, India.**

*E-mail: anjali.patel-chem@msubaroda.ac.in

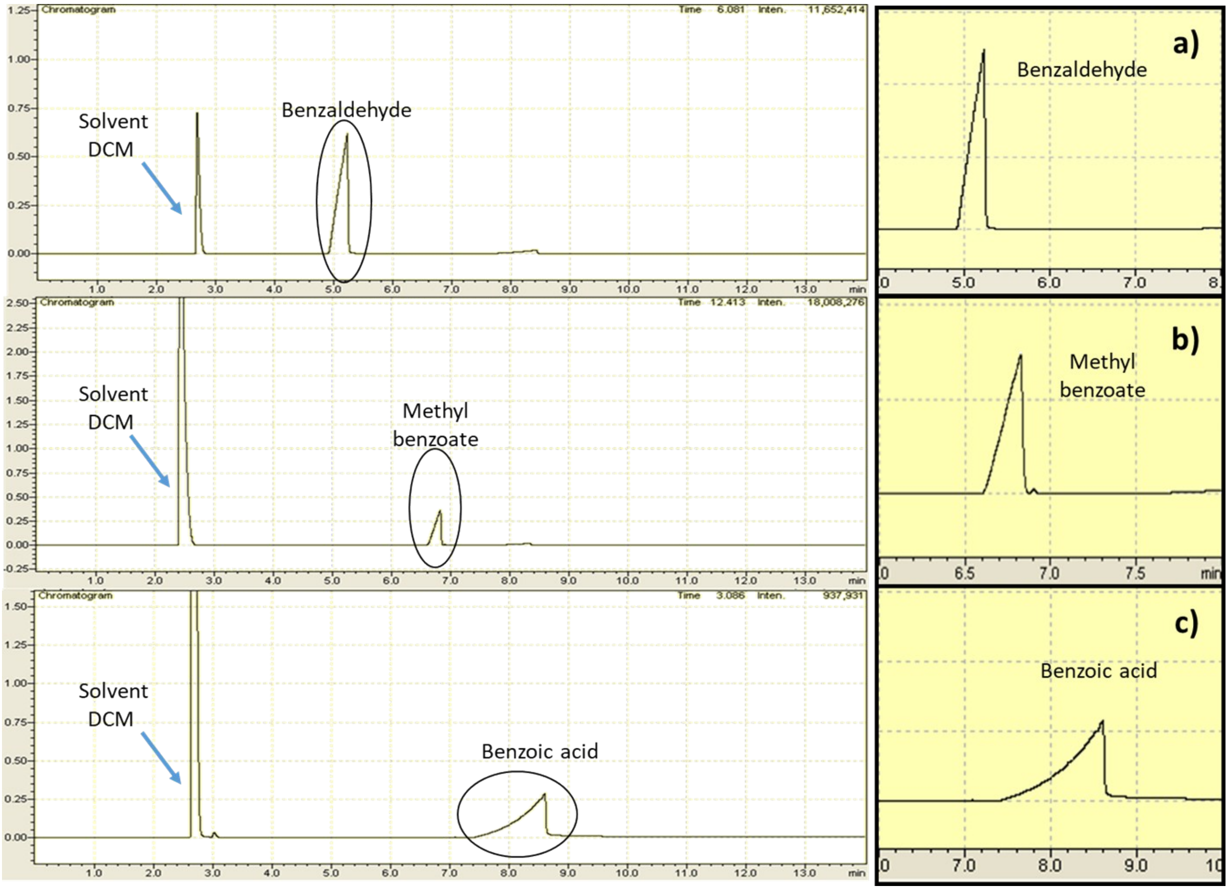

Figure 1S. GC Profile of stander a) DCM + benzaldehyde b) DCM + Methyl benzoate c) DCM + benzoic acid

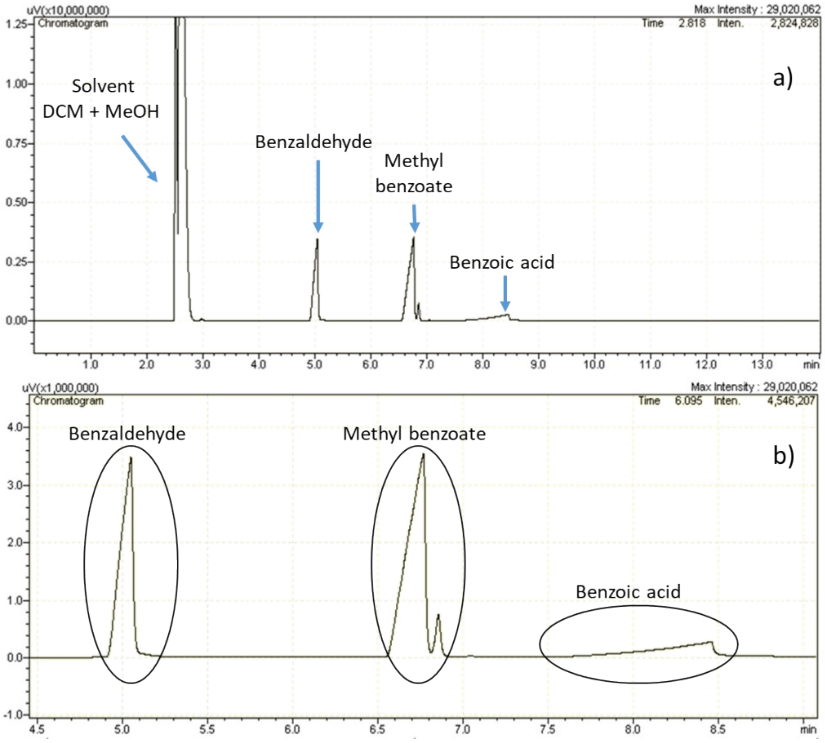

Figure S2. GC Analysis profile optimized conditions

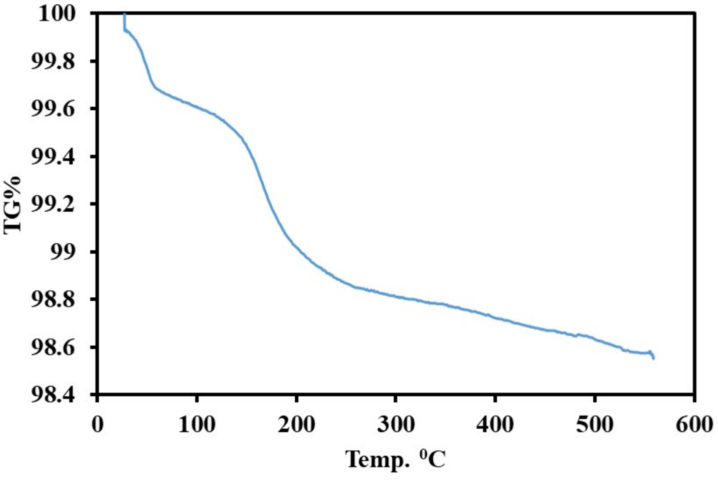

Figure S3. TGA of NiHPMA

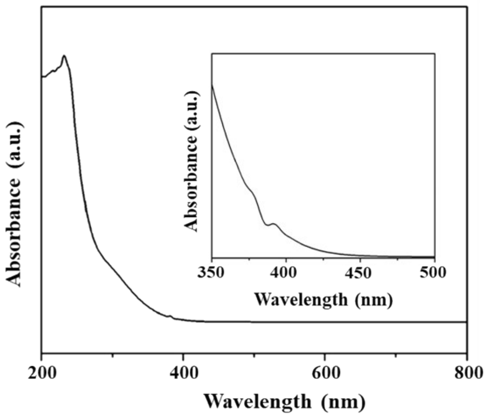

Figure S4. UV-Visible spectra of NiHPMA
